# Progress in Ophthalmology

**Published:** 1902-11-15

**Authors:** 


### Progress in Ophthalmology.

**Spontaneous Cure of Senile Cataract.**—Sydney Stephenson[1] quotes a case in which there was spontaneous cure of senile cataract in both eyes. It is refreshing to find in these days—when one hears every now and then of cases in which the cataract had cured itself after the medical man had pronounced the case incurable unless by operation—an authentic account like the present of how this cure came about. The patient was a highly intelligent lady of 55. He first saw her when her sight was beginning to fail, the right lens was opaque in its deeper parts, with a posterior polar opacity. The left lens had opaque striæ, and also a posterior polar opacity. Eight years later he saw her again, and she told the following story :—For several years she had been blind with her left eye, and able to see print with the right only by holding the book or paper close to her face. One afternoon, a fortnight before her visit, whilst at tea, she found that with her blind eye (left) she could see the tea-cups, and pattern on the tray. On the following day she saw the cornice of her sitting-room for the first time for several years. The sight of this eye continued to clear at the top, but a crescentic mass remained at the bottom of the field of vision. Four days later she could see the clergyman at church when he was in the pulpit. Four days after this, when going upstairs, the crescentic mass suddenly disappeared, and soon afterwards she could distinguish the lettering on passing omnibuses. On examination the vision of this left eye proved to be two-thirds of normal with a + 10 D lens, and with + 14 D she could read the smallest type on Jaeger's test card. The anterior chamber was deep, the pupil clear and black, the iris sluggish and tremulous, the tension normal. The dislocated lens was seen with the ophthalmoscope lying at the lower part of the vitreous chamber. The right lens at this time was seen to be opaque, the cataract there being nearly mature. Two years later she came again. Twelve days previous to her visit she noticed the sight of the right beginning to improve, so much so, that she could distinguish her husband across the room. For the next few days the eye was somewhat uncomfortable, mainly because there was a sort of "wobbling," and prismatic colours were visible when the electric light was used at night. Two days after this first improvement, while at dinner this time, she suddenly discovered that the sight of her right eye had become quite clear. On examination the same state of things, as in the left eye, was discovered ; the lens dislocated into the lower part of the vitreous, vision with +10 was again $\frac{2}{3}$ normal and with +12 the smallest print could be read. This, of course, was a very remarkable state of things, that in an old person both lenses should spontaneously dislocate themselves without the least injury or effort having taken place, and unfortunately cannot be hoped for unless in extremely rare cases. The only other ways that spontaneous cure of cataract can take place are, he says, by a slow absorption of the lens, a process that may seem to extend even to the capsule, according to several continental observers, or where the cataract takes on a morgagnian character, a condition in which the hardened nucleus floats in a liquefied cortex, the whole being enclosed in the lens capsule. Fuchs points out that the milky fluid cortex may actually be converted into a clear, transparent fluid, through which the patient may be able to distinguish external objects, especially where the nucleus lies out of the line of sight as it would do in the upright position.

**Rupture of the Sclerotic.**—Treacher Collins[3] showed a case of rupture of the sclerotic, caused by a cow's horn, at the Medical Graduates' College and Polyclinic. In commenting on these ruptures he pointed out that the thickest portion of the sclerotic is at the posterior part around the optic nerve, gradually tapering in passing forwards to the equator, where it is thinnest. A little past the equator the sclerotic receives an accession of fibres from the recti muscles, and again becomes thinner on approaching the corneal margin. At the equator, where the sclerotic is thinnest, it is supported by the tendons of the recti and the oblique muscles externally, and by the choroid and retina internally. The thinnest portion then of the sclerotic where it is not supported is a short distance from the sclero-corneal margin, about 3 mm. external to it. It is in this position where we meet with rupture of the sclerotic, when tension of the eye is suddenly increased from external pressure. The rupture extends into the extreme periphery of the anterior chamber, and the vitreous chamber is not opened unless the suspensory ligament is also torn and the lens displaced. He points out that the rupture is generally upwards, or upwards and inwards, as the violence comes either from below or below and on the outer side—the other sides are protected by the brow and nose. In the particular case he showed this was not so, the blow having been delivered from the lower and inner side of the globe and the rupture being up and out. We suppose the curly nature of the cow's horn must have had something to do with this unusual situation. Injuries such as this are not infrequent amongst those engaged in agricultural pursuits. Fuchs in his text book mentions the case of a man who had both his eyes ruptured by cows' horns, with an interval of two years. In his case both lenses were dislocated under the conjunctivæ, and it is interesting to observe that notwithstanding the violence—and it takes a good deal of violence to rupture a sclerotic—the conjunctiva frequently escapes injury, or at any rate actual tearing. Treacher Collins while commenting on the extraordinary bad luck of this man, was informed by an American surgeon, that he had had a case somewhat similar unless that the first eye was ruptured by the horn and the second eye by the tail of the same cow.

**Infantile Ophthalmia.**—Treacher Collins,[4] as a summary of a most exhaustive paper on the treatment of this disease, in which he quotes statistics from the large maternity hospitals of Great Britain, Ireland, and several of the Continental cliniques, advises that the following measures, which at the present time seem best calculated to reduce the amount of blindness, be adopted :—

1. Compulsory notification of cases of ophthalmia monatorium by all persons, attending women in labour, other than medical men.

2. Instruction as to the importance of the universal adoption of prophylactic measures (preferably Credé's method, 2 per cent. sol. nitrate of silver, the use of a sublimate solution, 1 in 2,000, or protargol 20 per cent.) by all lecturers and writers of text-books on midwifery.

3. The appointment of ophthalmic surgeons to maternity institutions, more especially those which provide for attendance of women at their own homes.

4. The provision in all midwifery bags of a drop bottle labelled "drops for the eyes."

5. The better training of monthly nurses in the methods of aseptic cleanliness.

[1] Lancet, April 26, 1902. [2] Lancet, April 12, 1902. [3] Clin. Jour., July 2, 1902 [4] Practitioner, April 1902.

(*To be continued.*)

